# Prediction of Intracranial Pressure in Patients with an Aneurysmal Subarachnoid Hemorrhage Using Optic Nerve Sheath Diameter via Explainable Predictive Modeling

**DOI:** 10.3390/jcm13072107

**Published:** 2024-04-04

**Authors:** Kwang Hyeon Kim, Hyung Koo Kang, Hae-Won Koo

**Affiliations:** 1 Clinical Research Support Center, Inje University Ilsan Paik Hospital, Goyang 10380, Republic of Korea; kh.kim@paik.ac.kr; 2 Division of Pulmonary and Critical Care Medicine, Department of Internal Medicine, Inje University Ilsan Paik Hospital, Inje University College of Medicine, Goyang 10380, Republic of Korea; inspirit26@paik.ac.kr; 3 Department of Neurosurgery, College of Medicine, Inje University Ilsan Paik Hospital, Goyang 10380, Republic of Korea

**Keywords:** optic nerve sheath diameter, intracranial pressure, cerebral aneurysm, ultrasound, explainable artificial intelligence

## Abstract

**Background:** The objective of this investigation was to formulate a model for predicting intracranial pressure (ICP) by utilizing optic nerve sheath diameter (ONSD) during endovascular treatment for an aneurysmal subarachnoid hemorrhage (aSAH), incorporating explainable predictive modeling. **Methods:** ONSD measurements were conducted using a handheld ultrasonography device during the course of endovascular treatment (*n* = 126, mean age 58.82 ± 14.86 years, and female ratio 67.46%). The optimal ONSD threshold associated with an increased ICP was determined. Additionally, the association between ONSD and ICP was validated through the application of a linear regression machine learning model. The correlation between ICP and various factors was explored through the modeling. **Results:** With an ICP threshold set at 20 cmH_2_O, 82 patients manifested an increased ICP, with a corresponding ONSD of 0.545 ± 0.08 cm. Similarly, with an ICP threshold set at 25 cmH_2_O, 44 patients demonstrated an increased ICP, with a cutoff ONSD of 0.553 cm. **Conclusions:** We revealed a robust correlation between ICP and ONSD. ONSD exhibited a significant association and demonstrated potential as a predictor of ICP in patients with an ICP ≥ 25 cmH_2_O. The findings suggest its potential as a valuable index in clinical practice, proposing a reference value of ONSD for increased ICP in the institution.

## 1. Introduction

Increased intracranial pressure (IICP) is characterized by an intracranial pressure (ICP) exceeding 15–20 mmHg for more than 5 min in unstimulated patients [1]. The accurate prediction of IICP is crucial in the management of individuals with brain lesions, as it can lead to brain herniation, posing a significant risk of irreversible brain damage and mortality [2]. The American Brain Trauma Foundation recommends ICP monitoring for all cases of traumatic brain injury with a Glasgow Coma Scale score of 3–8 and abnormal findings on computed tomography (CT) scans, including hematomas, contusions, severe swelling, herniation, and basal cistern collapse [3].

Various invasive and noninvasive methods and devices are employed for ICP measurement [4]. Direct evaluation of IICP involves measuring ICP values in different anatomical spaces by placing devices into the intraventricular, intraparenchymal, epidural, subdural, and subarachnoid spaces [2,4]. However, this technique is invasive, requires neurosurgical expertise, and repeat ICP measurements present challenges.

Recently, noninvasive techniques for measuring and predicting ICP in patients with altered consciousness levels have emerged [2]. Among these, the optic nerve sheath diameter (ONSD) serves as a valuable indirect diagnostic method for detecting changes in ICP [5,6,7,8]. Helmke and Hansen initially reported a correlation between ICP values and ONSD measured through linear ultrasonography (US) [9]. The optic nerve is enveloped by the arachnoid membrane, containing cerebrospinal fluid (CSF). In the case of IICP, the ONSD may increase due to the filling of the subarachnoid space with CSF [10].

An investigation was executed employing artificial intelligence (AI) to anticipate alterations within intervals of several tens of minutes, with the aim of identifying abnormal fluctuations in ICP among individuals afflicted with traumatic brain injury. Ye et al. employed a long short-term memory (LSTM) model to analyze data sourced from an open database. In terms of predictive performance assessment, the outcomes revealed an average accuracy of 94.62%, an average sensitivity of 74.91%, an average specificity of 94.83%, and an average root mean square error of approximately 2.18 mmHg [11]. Miyagawa conducted an assessment of optic nerve sheath diameter (ONSD) utilizing computed tomography (CT) images in pediatric cases where heightened intracranial pressure was suspected [12]. Subsequently, an endeavor was made to forecast ICP, employing machine learning methodologies. It was observed that both the mean ONSD and the ventricular width exhibited statistical significance in children displaying indicators of increased ICP.

AI techniques hold significant potential in predicting clinical outcomes, encompassing areas such as disease diagnosis, treatment response, and patient survival [13,14,15]. Nonetheless, these techniques also have certain limitations. On the positive side, AI offers the capability to process vast amounts of data, uncovering intricate patterns that may be challenging for humans to discern. This ability enhances the accuracy of clinical outcome predictions. Additionally, AI algorithms excel in analyzing data at a much faster pace than humans, thereby expediting the diagnosis and treatment of patients.

Concurrently, the introduction of the concept of explainable artificial intelligence (XAI) has emerged as a solution to address the challenges associated with interpreting the results generated by AI [16]. Given the inherently opaque nature of AI models, which function as black box models, the integration of XAI marks the commencement of active research in the field of neurosurgery [17,18,19]. Nevertheless, there is a scarcity of published results from AI analyses specifically predicting ICP using optic nerve sheath diameter (ONSD). Furthermore, studies aiming to enhance result interpretability by incorporating explainable artificial intelligence (XAI) into these analyses are limited.

The primary objective of this investigation was to construct a predictive model for intracranial pressure (ICP) utilizing the optic nerve sheath diameter (ONSD) measured through ultrasonography (US) during endovascular treatment for an aneurysmal subarachnoid hemorrhage (aSAH), incorporating explainable artificial intelligence (XAI).

## 2. Materials and Methods

### 2.1. Study Population and Data Collection

This study constitutes a retrospective investigation conducted at a singular medical center, involving the analysis of prospectively gathered data from a database encompassing patients diagnosed with an aneurysmal subarachnoid hemorrhage (aSAH). Systematic electronic searches within the database were executed, revealing a cohort of 126 consecutive aSAH patients admitted to our hospital between January 2018 and April 2022. Inclusion criteria comprised (1) individuals aged >18 years, (2) subjected to endovascular treatment (coiling), (3) with pre-treatment confirmation of optic nerve sheath diameter (ONSD) through ultrasound, and (4) subsequent lumbar puncture for ICP evaluation post-treatment. Exclusions were made for (1) patients lacking ONSD data due to prior ocular surgery and (2) those undergoing lumbar puncture for ICP determination, resulting from prior spinal surgery. The measurement of ONSD using US and ICP using lumbar puncture are conducted almost simultaneously.

Demographic characteristics and vascular risk factors of aSAH patients were meticulously scrutinized, encompassing parameters such as age at admission, gender, history of hypertension, diabetes mellitus, hyperlipidemia, smoking and alcohol habits, aneurysm dimensions (longest diameter) and location, as well as the Hunt–Hess grade and Fisher grade upon admission. Hypertension was delineated based on antecedent diagnosis and the utilization of antihypertensive medications.

Upon transfer of patients to the intensive care unit for postoperative management, lumbar puncture was conducted under general anesthesia using a 20 g spinal needle to gauge ICP. The opening pressure during lumbar puncture was quantified in centimeters of water (cmH_2_O). IICP was characterized as being equal to or exceeding either 20 or 25 cm of water (cmH_2_O).

Approval for this study, encompassing the review and dissemination of information gleaned from patient records, was granted by the Institutional Review Board of Ilsan Paik Hospital (IRB approval no.: 2021-10-017-001).

### 2.2. Measurement of Optic Nerve Sheath Diameter

As a single neurosurgeon may not be available daily, decisions are reached through measurements and discussions between two experienced neurosurgeons. The ONSD was measured only once, immediately after endovascular treatment, where both the left and right ONSD were assessed and the mean value of the two measurements was employed. Subsequently, upon uploading the ultrasound images to the picture archiving and communication system (PACS), two neurosurgeons engaged in discussions regarding the measurements. The handheld US device utilized for measurement was the Vscan (GE Healthcare, New York, NY, USA), employing a broad-bandwidth linear array probe with a frequency range of 3.3–8.0 MHz. ONSD measurements were taken with the patient in a supine position under general anesthesia. To safeguard against corneal injury, soft transparent silicone tapes were affixed to the bilateral periorbital skin area. The Vscan Extend US device, featuring an ophthalmic mode, was employed. Subsequent to obtaining ONSD images for both orbits, the acquired US images were transmitted to the picture archiving communication system (PACS). The ONSD measurements, expressed in centimeters, were taken at a depth of 3 mm behind the eye globe [10]. The calculated ONSD value represented the mean of measurements obtained from both orbits.

### 2.3. Research Diagram

**Figure 1:**
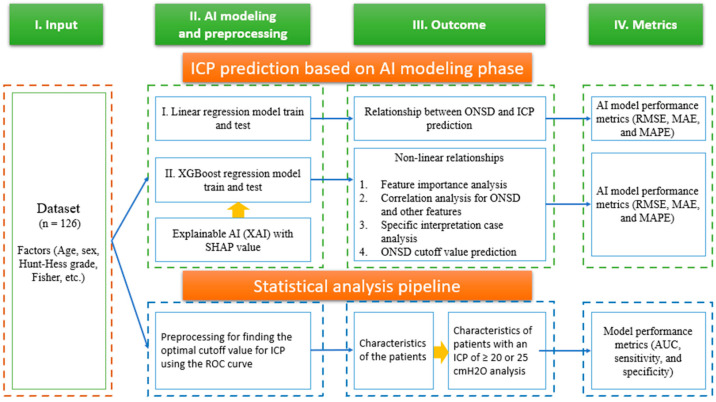
Research architecture including ICP prediction based on AI modeling and basic statistical analysis for this study.

Figure 1 illustrates the research flow chart for this study. In the initial stage, a dataset containing prognostic factors, such as age, sex, Hunt–Hess score, Fisher grade, optic nerve sheath diameter (ONSD), and ICP information, was gathered for 126 patients. Following data preprocessing, involving bias and outlier rejection, a linear regression model was employed for predictive modeling. The receiver operating characteristic curves and the corresponding area under the curve were computed to ascertain the optimal ONSD cutoff value associated with increased ICP.

Subsequently, the Shapley additive explanation (SHAP) was utilized to articulate the interpretability for the non-linear relationship between prognostic factors of the modeling techniques using XGBoost regression. Finally, the correlation between ONSD and ICP was modeled. Furthermore, the significance of each prognostic factor and the predictive outcomes of the key prognostic factors were graphically represented. Also, the correlation for ONSD and other factors, specific patients’ interpretation, and ONSD cutoff value prediction were analyzed (Figure 1). The model’s performance was assessed through metrics including root mean square error (RMSE), mean absolute error (MAE), and mean absolute percentage error (MAPE).

In the subsequent stage, a fundamental demographic analysis of the included individuals, named Hamzas, was conducted and statistical significance was confirmed by categorizing the ICP values into 20 and 25 cmH_2_O segments. Chi-Square tests were employed for the analysis of categorical variables, while independent *t*-tests were utilized for the examination of continuous variables. Additional analysis was conducted to determine the optimal ONSD cutoff value based on the ICP reference value with the maximum area under the curve (AUC) (statistical significance: *p* < 0.05).

### 2.4. Programming Environment and Analysis Software Used

The computational setting utilized encompassed Python version 3.9, along with the inclusion of scikit-learn (version 1.0.2), statsmodel (version 0.13.2), and shap (version 0.42.1) libraries. The statistical analysis was performed using SPSS 21.0 for windows (SPSS Inc., Chicago, IL, USA)

## 3. Results

### 3.1. Clinical Characteristics of the Patients and the Criteria for ICP > 20 and 25 cmH_2_O

A total of 126 consecutive patients diagnosed with an aneurysmal subarachnoid hemorrhage (aSAH) fulfilled the inclusion criteria. The baseline characteristics of the patients and aneurysms are presented in Table 1. The average age of the participants was 58.82 years, with an average aneurysm size of 6.93 mm. The optic nerve sheath diameter (ONSD) averaged 0.54 cm and the mean ICP measured through lumbar puncture was 22.45 cmH_2_O.

Table 2 delineates the clinical features of patients exhibiting IICP equal to or exceeding 20 cmH_2_O. Notably, the mean ICP for the subset of 67 patients with IICP ≥ 20 cmH_2_O was 29.59 cmH_2_O (*p* = 0.000). These patients demonstrated a correlation with poorer outcomes, as evidenced by Hunt–Hess grade (*p* = 0.000) and Fisher grade (*p* = 0.045). The mean ONSD in this group measured 5.96 mm (*p* = 0.000).

Table 3 presents the clinical characteristics of individuals exhibiting IICP equal to or exceeding 25 cmH_2_O. Furthermore, the average ICP among the cohort of 44 patients with IICP ≥ 25 cmH_2_O was determined to be 33.84 cmH_2_O (*p* = 0.000). These individuals were predominantly situated within a cohort characterized by a more unfavorable distribution in terms of Hunt–Hess grade (*p* = 0.000) and Fisher grade (*p* = 0.024). The mean ONSD was measured at 0.61 cm (*p* = 0.000).

### 3.2. Association between ONSD and ICP

**Figure 2:**
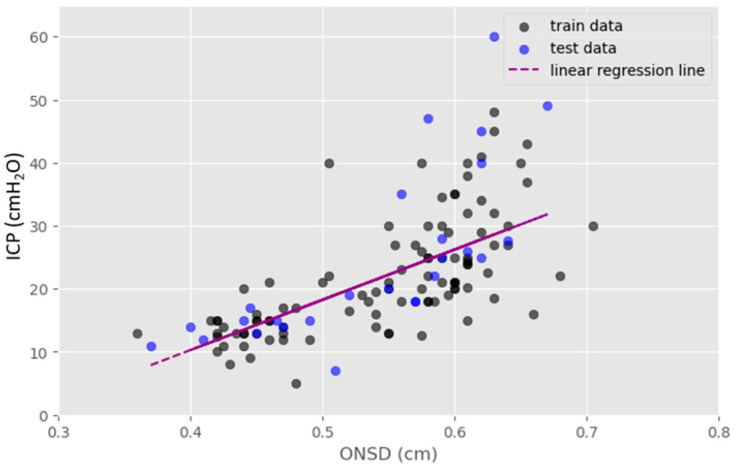
Linear regression was employed to explore the association between ONSD and ICP. The resulting linear relationship equation is expressed as follows: ICP (cmH_2_O) = 79.70 × ONSD (cm) − 21.60.

The ONSD exhibited a linear correlation with ICP, as determined through linear regression analysis utilizing the linear regression model (Figure 2). The mean ONSD data demonstrated a uniform distribution density ranging from 0.35 to 0.7 cm and the results of ICP prediction followed a linear trend. In Figure 2, the dark green dots represent the training dataset, while the blue dots represent the test dataset. The linear regression analysis generated a line of best fit described using the following equation: ICP (cmH_2_O) = 79.70 × ONSD (cm) − 21.60. The coefficient of determination is 0.54 in the model.

### 3.3. Global Feature and Correlation Analysis for the Risk Factors Related to ICP

**Figure 3:**
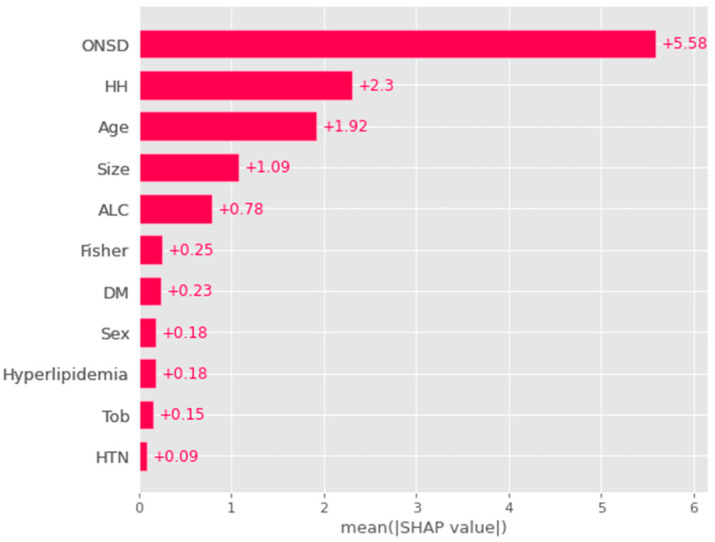
An analysis of the features associated with the risk factors of ICP revealed that ONSD was the most significant factor correlated with ICP. This was followed by Hunt–Hess grade, age, and aneurysm size. The feature importance was elucidated through a global method, specifically by averaging contributions for all patients using the SHAP (SHapley Additive exPlanations) explainer. Note: ONSD = optic nerve sheath diameter; HTN = hypertension; DM = diabetes mellitus; ALC = alcohol, HH = Hunt–Hess grade, Fisher = Fisher grade; Size = aneurysm size; Tob = tobacco.

Figure 3 illustrates the factors influencing ICP, based on their respective importance. ONSD demonstrated the closest association with ICP, with a mean SHAP value of 5.58. Following ONSD, the factors of significance included Hunt–Hess grade (mean SHAP: 2.3), age (mean SHAP: 1.92), and aneurysm size (mean SHAP: 1.09). The mean SHAP values represent the average contribution for the dependent variable, ICP, across the entire patient dataset. Consequently, these results denote the global feature importance (Figure 3).

**Figure 4:**
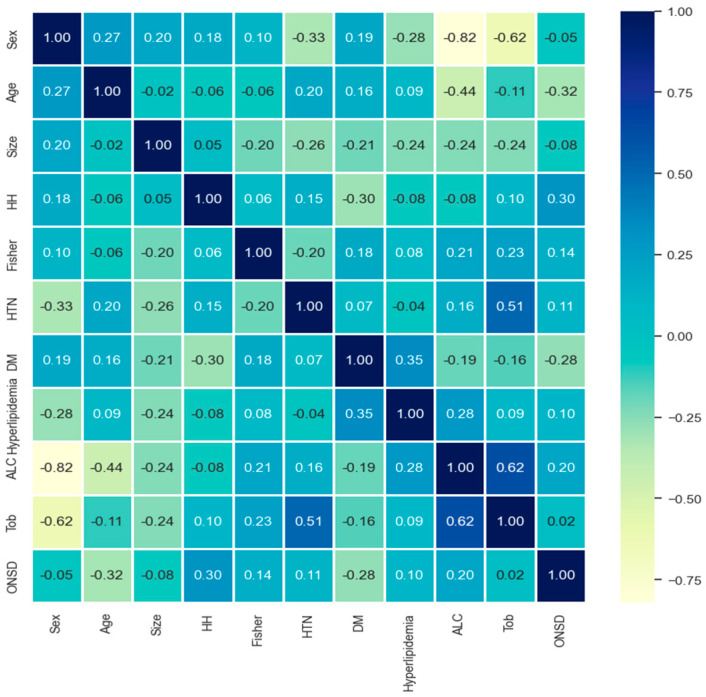
Correlation analysis for prediction model (absolute value of the correlation coefficient: 0.8–1.0 highly, 0.6–0.8 strong, 0.4–0.6 medium, 0.2–0.4 weak, 0.0–0.2 weak or no correlation). Note: HH = Hunt–Hess grade, Fisher = Fisher grade; HTN = hypertension; DM = diabetes mellitus; ALC = alcohol abuse; Tob = tobacco, smoking behavior; ONSD = optic nerve sheath diameter.

The correlation between various factors was examined (Figure 4). Factors of the alcohol and tobacco exhibited a robust correlation coefficient of 0.62, while hypertension and tobacco displayed a moderately high correlation of 0.51. Conversely, for women, alcohol consumption and smoking demonstrated a very strong negative correlation of −0.82 and −0.62, respectively. However, a weak correlation was observed between ONSD and other neurological factors.

### 3.4. Individual SHAP Analysis Result in Each Patient

**Figure 5:**
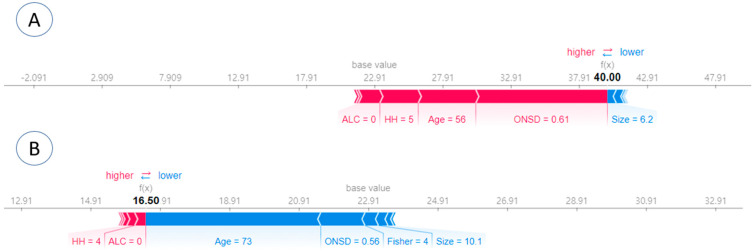
Local interpretation was achieved through individual SHAP analyses for each patient. (**A**) The prediction was conducted with an ICP of 40 cmH_2_O, yielding a SHAP ICP score of 40. Notably, a Hunt–Hess grade of 5 and an ONSD of 0.61 exerted a substantial influence, indicating a high ICP value. (**B**) The prediction was made with an ICP of 18 cmH_2_O, resulting in a SHAP ICP score of 16.50. In this scenario, a Hunt–Hess grade of 4 positively impacted the prediction, while age had a minimal negative impact.

Individual SHAP analyses were conducted for local interpretation, as depicted in Figure 5. In case A, the prediction was made with an ICP of 40 cmH_2_O and a SHAP ICP score of 40 was obtained. Concurrently, a Hunt–Hess grade of 5 and an ONSD of 0.61 were identified to have a significant influence, suggesting a high ICP value (Figure 5A). In case B, the prediction was carried out with an ICP of 18 cmH_2_O and a SHAP ICP score of 16.50. Consequently, a Hunt–Hess grade of 4 had a positive impact, while age had a minimal negative impact (Figure 5B).

### 3.5. ICP Prediction Explainer for Criteria ≥ 25 cmH_2_O

**Figure 6:**
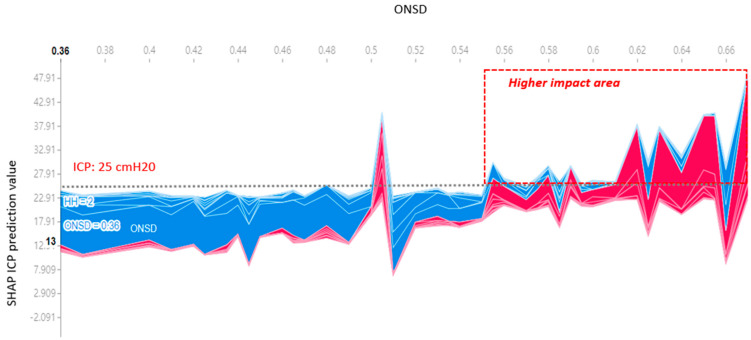
The change in impact of ONSD in predicting ICP using the SHAP explainer is evident. As the ONSD width increases beyond 0.55 cm, there is a corresponding rise in ICP, elevating the SHAP value above the mean ICP of 25 cmH_2_O. Note: The highness or lowness of ICP prediction values = Red for high and blue for low.

There was a linear proportional relationship observed between ONSD and ICP (Figure 2). Specifically, an ONSD equal to or greater than 0.56 cm and a mean ICP equal to or greater than 25 cmH_2_O significantly influenced the SHAP value in Figure 6. This outcome shows a consistent prediction output aligned with the model derived from linear regression.

### 3.6. ONSD Cutoff Values and Performance Metrics

When the threshold for IICP was established at ≥20 cmH_2_O, the ONSD cutoff value was determined to be 0.545 cm, exhibiting a sensitivity of 92.5% and a specificity of 78.0%. In cases where the IICP threshold was increased to ≥25 cmH_2_O, the optimal ONSD cutoff value shifted to 0.553 cm, demonstrating a sensitivity of 95.5% and a specificity of 67.1% (Table 4).

Table 5 presents the root mean square error (RMSE), mean absolute error (MAE), and mean absolute percentage error (MAPE) values for both the linear regression and XGBoost regression models. The RMSE values for linear regression and XGBoost were recorded as 2.64 and 2.74, respectively, while the corresponding MAPE values were 0.36 and 0.42. These outcomes suggest a low error rate, each falling below 0.5.

## 4. Discussion

### 4.1. The Association between ONSD and IICP

Typical intracranial pressure (ICP) exhibits a variability associated with age and body posture. However, the generally accepted range for individuals in the supine position is 7.5 to 20 cmH_2_O in healthy adults [2]. In this investigation, the criteria for increased ICP (IICP) were delineated at 20 and 25 cmH_2_O, considering diverse reference values reported in previous studies [3,20,21].

During brain surgery, the surgeon can visualize the optic nerve sheath, which is visually blocked by the adjacent cranial nerves (CN II), optic chiasm, and internal carotid artery (ICA), and identify abnormal enlargements [22]. It is based on visual assessment rather than precise measurements.

Within this study, optic nerve sheath diameter (ONSD) measurements, obtained using a handheld ultrasound (US) device, demonstrated a robust linear correlation with ICP directly measured through lumbar puncture. Consequently, the presence and magnitude of IICP can be deduced and IICP values can be predicted by assessing ONSD via a handheld US device, eliminating the need for invasive ICP measurement. When considering reference IICP values of 20 and 25 cmH_2_O, corresponding ONSD cutoff values were affirmed at 5.45 and 5.53 mm, respectively. The marginal deviation from the empirically measured cutoff value, within the context of the error range, suggests clinical significance in using an IICP threshold exceeding 5.5 mm for patient treatment decisions.

Various noninvasive techniques, such as transcranial Doppler US, tympanic membrane displacement, and US, have been employed for IICP detection. Among these, US proves efficient and easily learnable, with low intra- and inter-observer variations and minimal technical complexity [23,24,25,26]. Since its introduction in 1996 by Helmke and Hansen, transorbital sonography has been continuously evaluated for ICP assessment [9]. Despite its utility in emergency cases and real-time IICP changes prediction, the technique requires ongoing validation.

The optic nerve sheaths, contiguous with the brain and formed by the dura mater, allow for cerebrospinal fluid (CSF) movement through subarachnoid spaces within intracranial regions. Subarachnoid spaces expand in intra-orbital areas and IICP leads to CSF shifting into the optic nerve sheath (ONS), resulting in anterior ONS enlargement due to CSF accumulation [9,23,26]. Therefore, ONSD is generally assessed 3 mm behind the optic globe.

ONSD variations based on ethnicity and genetics introduce a potential confounding factor in establishing an optimal ONSD for defining IICP. Numerous studies report varying typical ONSD values across different populations [27,28,29,30,31,32,33]. In Western countries, the typical ONSD values are 2.4–4.7 mm in the United Kingdom [34], 4.3–7.6 mm in Germany [27,28], and 4.5–7.7 in Italy [29]. In Asian populations, the typical ONSD values are 4.24–4.83 mm in Bangladesh [30], 2.65–4.30 in China [31], 2.6–4.1 mm in Iran [32], and 3.3–5.2 mm in the Republic of Korea [33].

Despite several studies exploring the correlation between ONSD and IICP, consensus on the optimal ONSD cutoff value for IICP diagnosis remains elusive [25,35].

Tayal documented a correlation between IICP observed on brain CT scans and a mean ONSD of 6.27 mm, associating it with an established ONSD cutoff value of 5.54 mm [25]. In Blaivas’ investigation, there was an observed correlation between IICP on brain CT scans and a mean ONSD of 6.27 mm, with a designated ONSD cutoff value of 5.6 mm. [35]. Multiple studies have suggested specific ONSD cutoff values, such as 5.86 mm, as proposed by Geeraerts [36]. The suggested ONSD cutoff values include 5.7 mm, according to Soldatos [37], and 5.5 mm based on Amini’s research [38].

Blaivas et al. initially demonstrated a strong correlation between an enlarged ONSD observed through ocular ultrasound and IICP [35]. Due to numerous studies focusing on IICP-related ONSD measurements obtained through US, an acknowledged IICP cutoff value of 5.2 mm has been established [33,36,37,38,39]. Nevertheless, the determination of the cutoff value involves consideration of various factors. These may include ethnicity, the methodology employed for ICP measurement, as well as the location and volume of the hematoma [24,37]. In a prior investigation involving a Korean population with moderate hematoma, the identified optimal ONSD cutoff value for detecting IICP was 5.6 mm [23]. If the IICP was >20 cmH_2_O, a mean ONSD value of 5.96 mm and an ONSD cutoff value of 5.45 mm were correlated with IICP. And if the IICP was 25 cmH_2_O, a mean ONSD value of 6.10 mm and an ONSD cutoff value of 5.53 were correlated with IICP.

Intracranial hypertension significantly impacts the association between ONSD and IICP [7,23,24,36,40,41]. Nevertheless, the extent to which its impact is accurately represented in patients with aSAH has been a matter of controversy. Yesilaras noted that evaluating the ONSD on a head CT scan conducted due to suspected spontaneous SAH could potentially aid in the diagnosis of spontaneous SAH [6]. Furthermore, according to Lee’s study, the ONSDs of individuals with aSAH exhibiting unfavorable neurological outcomes were notably larger compared to those with favorable neurological outcomes. Consequently, ONSD demonstrates prognostic significance in patients with aSAH [42]. For cases of Terson syndrome, the measurement of ONSD through US proves valuable for both diagnosis and monitoring the progression of IICP [39]. Nonetheless, recent studies have conveyed findings suggesting that ONSD may not accurately reflect IICP in patients with aSAH [43]. In Zoerle’s investigation, involving 20 patients with aSAH, ONSD measurements obtained through US exhibited weak correlation with ICP measured directly and simultaneously through ventricular catheters [43]. Bauerle’s study did not reveal any correlations between ONSD measured through US and IICP in a cohort of 27 patients with aSAH [21]. Two studies put forth two potential explanations. Firstly, the ONS demonstrated compromised retraction capacity following severe exposure to ICP exceeding 45 mmHg. Secondly, there is a restriction in CSF circulation along the ONS [21].

If neurosurgeons can efficiently, promptly, and precisely predict ICP at the bedside using a handheld US device, it opens the possibility for swift and targeted management. Additionally, such a capability would prove immensely valuable in the early differentiation of the underlying causes of alterations in mental function following treatment. This differentiation could determine whether the changes are attributable to IICP or if they stem from secondary causes such as medical issues and epilepsy.

### 4.2. Utilizing XAI for the Modeling of ICP

The utilization of modeling for ICP prediction using XAI offers several advantages. Firstly, it can serve as a clinical decision support system, aiding in the prognosis of patients with increased ICP by measuring ONSD. Additionally, AI algorithms have the potential to provide personalized treatment recommendations by analyzing individual patient data. The incorporation of local interpretation methods for each prognostic factor allows for a nuanced understanding of the impact of different factors on ICP in individual patient cases. Moreover, AI algorithms contribute to minimizing human errors, such as misinterpretation of data or overlooking key factors.

However, drawbacks exist in the use of AI techniques. Firstly, the accuracy of AI predictions relies on the quality of the data used for algorithm training. Incomplete or inaccurate data can lead to erroneous predictions. Secondly, AI algorithms may exhibit bias if training data are biased or if the algorithm fails to account for differences in patient populations. Thirdly, certain AI algorithms operate as black boxes, lacking the ability to explain the rationale behind specific predictions. This poses challenges for clinicians in comprehending the basis for predictions and making informed treatment decisions. Nevertheless, the integration of XAI techniques, such as the SHAP explainer (Figure 5 and Figure 6), addresses the black box issue by enhancing the explainability of correlations between ICP, ONSD, and other prognostic factors through both global and local interpretation (Figure 3 and Figure 4). The determined ONSD cutoff value was 0.55 cm, correlating with ICP values equal to or exceeding 25 cmH_2_O, as delineated in Table 4. This finding aligns with the outcomes of the SHAP analysis. In simpler terms, a notable impact zone manifested above the threshold of 0.55 cm, visually represented as the red area (Figure 6).

The present study is not without limitations. The small sample size and the single-institution setting could introduce bias into the dataset and potentially impact the generalizability of the prediction model (Table 1). Consequently, future studies with larger datasets and diverse patient populations are essential to mitigate potential biases.

While AI techniques offer considerable potential for improving clinical outcome predictions, their limitations should be approached with caution, such as global and local interpretation (Figure 3 and Figure 4) [44,45]. Clinical researchers and healthcare professionals must remain vigilant regarding potential biases and limitations associated with AI algorithms. Additionally, the responsible and transparent use of these algorithms is crucial to ensuring their benefits for patients and enhancing healthcare outcomes.

Our future research can prioritize studies with multicenter datasets, encompassing diverse patient populations. Additionally, exploring a wider range of XAI techniques like LIME and feature importance analysis could provide a more comprehensive understanding of the model’s decision-making process. Furthermore, evaluating the model’s generalizability across a broader spectrum of ICP values would offer a clearer picture of its effectiveness. External validation through testing on independent datasets from different institutions is crucial to solidify the model’s generalizability.

## 5. Conclusions

The measurement of optic nerve sheath diameter (ONSD) using portable ultrasound (US) devices holds significance in predicting and assessing intracranial pressure (ICP) in patients with brain lesions. Despite the utilization of clinical data from a relatively small cohort of Koreans from a single institution in this study, explainable artificial intelligence techniques were employed to discern the correlation between ICP and ONSD. Moreover, an attempt was made to conduct factor analysis to explore the non-linear relationship.

Our model suggests that the factors influencing ICP, in order of decreasing importance, are ONSD, Hunt–Hess grade, and age. We conducted individual SHAP analyses to provide local interpretations for specific patients. These analyses, along with the model itself, identified Hunt–Hess grade, age, and ONSD as having a significant influence on ICP, suggesting a high likelihood of increased ICP.

Notably, this study suggests that the presented findings could serve as valuable indicators in clinical practice. The provision of reference values and predicted values for ONSD specific to the Korean population with increased intracranial pressure (IICP) enhances the clinical utility of these results.

## Figures and Tables

**Table 1:** Characteristics of the patients and aneurysms.

Features	All Participants (*n* = 126)
Age (years)	58.82 ± 14.86
Sex [Female, *n* (%)]	85 (67.46)
Aneurysm size (mm)	6.93 ± 4.12
Aneurysm location (*n*, %)	
ACA	44 (34.92)
MCA	18 (14.29)
ICA	41 (32.54)
Posterior circulation	23 (18.25)
Hunt–Hess grade (*n*, %)	
1	0 (0.00)
2	55 (43.65)
3	38 (30.16)
4	28 (22.22)
5	5 (3.97)
Fisher grade (*n*, %)	
1	3 (2.38)
2	21 (16.67)
3	24 (19.05)
4	78 (61.90)
Hypertension (*n*, %)	59 (46.83)
Diabetes (*n*, %)	17 (13.49)
Hyperlipidemia (*n*, %)	24 (19.05)
Smoking (*n*, %)	33 (26.19)
Alcohol (*n*, %)	39 (30.95)
Optic nerve sheath diameter (cm)	0.545 ± 0.08
Intracranial pressure (cmH_2_O)	22.45 ± 10.28

Note: ACA = anterior cerebral artery; MCA = middle cerebral artery; ICA = internal carotid artery.

**Table 2:** Characteristics of patients with an ICP ≥ 20 cmH_2_O.

Characteristics	Patients with an ICP ≥ 20 cmH_2_O (*n* = 67)	Patients with an ICP < 20 cmH_2_O (*n* = 59)	*p*-Value
Age (years)	55.33 ± 13.73	62.78 ± 15.20	0.005
Sex [Female, *n* (%)]	41 (61.19)	44 (74.58)	0.110
Aneurysm size (mm)	6.74 ± 3.76	7.15 ± 4.53	0.579
Aneurysm location (*n*, %)			0.09
ACA	28 (41.79)	16 (27.12)	
MCA	12 (17.91)	6 (10.17)	
ICA	18 (26.87)	23 (38.98)	
Posterior circulation	9 (13.43)	14 (23.73)	
Hunt–Hess grade			0.000
1	0 (0.00)	0 (0.00)	
2	19 (28.36)	36 (61.02)	
3	21 (31.34)	17 (28.81)	
4	23 (34.33)	5 (8.47)	
5	4 (5.97)	1 (1.69)	
Fisher grade			0.045
1	0 (0.00)	3 (5.08)	
2	8 (11.94)	13 (22.03)	
3	11 (16.42)	13(22.03)	
4	48 (71.64)	30 (50.85)	
Hypertension (*n*)	30 (44.78)	29 (49.15)	0.623
Diabetes (*n*)	7 (10.45)	10 (16.95)	0.286
Hyperlipidemia (*n*)	12 (17.91)	12 (20.34)	0.729
Smoking (*n*)	17 (25.37)	16 (27.12)	0.824
Alcohol (*n*)	22 (32.84)	17 (28.81)	0.626
Optic nerve sheath diameter (cm)	0.596 ± 0.046	0.486 ± 0.067	0.000
Intracranial pressure (cmH_2_O)	29.59 ± 8.97	14.35 ± 3.08	0.000

**Table 3:** Characteristics of patients with an ICP ≥ 25 cmH_2_O.

Characteristics	Patients with an ICP ≥ 25 cmH_2_O (*n* = 44)	Patients with an ICP < 25 cmH_2_O (*n* = 82)	*p*-Value
Age (years)	54.20 ± 14.11	61.29 ± 14.72	0.010
Sex [Female, *n* (%)]	24 (54.55)	61 (74.39)	0.023
Aneurysm size (mm)	6.63 ± 3.44	7.10 ± 4.46	0.546
Aneurysm location (*n*, %)			0.021
ACA	21 (47.73)	23 (28.05)	
MCA	9 (20.45)	9 (10.98)	
ICA	9 (20.45)	32 (39.02)	
Posterior circulation	5 (11.36)	18 (21.95)	
Hunt–Hess grade			0.000
1	0 (0.00)	0 (0.00)	
2	7 (15.91)	48 (58.54)	
3	16 (36.36)	22 (26.83)	
4	17 (38.64)	11 (13.41)	
5	4 (9.09)	1 (1.22)	
Fisher grade			0.024
1	0 (0.00)	3 (3.66)	
2	4 (9.09)	17 (20.73)	
3	5 (11.36)	19 (23.17)	
4	35 (79.55)	43 (52.44)	
Hypertension (*n*)	21 (47.73)	38 (46.34)	0.882
Diabetes (*n*)	4 (9.09)	13 (15.85)	0.289
Hyperlipidemia (*n*)	10 (22.73)	14 (17.07)	0.441
Smoking (*n*)	11 (25.00)	22 (26.83)	0.824
Alcohol (*n*)	17 (38.64)	22 (26.83)	0.172
Optic nerve sheath diameter (cm)	0.61 ± 0.035	0.51 ± 0.075	0.000
Intracranial pressure (cmH_2_O)	33.84 ± 8.28	16.34 ± 4.20	0.000

**Table 4:** ONSD cutoff values were determined based on ICP values. AUC, sensitivity, and specificity assessments were obtained through statistical analysis.

ICP (cmH_2_O)	AUC	95% CI	ONSD Cutoff Value (cm)	Sensitivity (%)	Specificity (%)	PPV (%)	NPV (%)
≥20	0.90	0.85–0.96	0.545	92.50	78.00	82.70	90.20
≥25	0.87	0.81–0.93	0.553	95.50	67.10	60.90	96.50

**Table 5:** Performance metrics for the linear regression and XGBoost regression models.

Model	RMSE	MAE	MAPE
Linear regression	2.64	5.77	0.36
XGBoost regression	2.74	6.26	0.42

## Data Availability

All clinical data collected will be used exclusively for this research study, in accordance with IRB regulations. There will be no disclosure, transfer, or sharing of this data for any other purpose.
